# Abemaciclib: A multi-functional radiation modifier

**DOI:** 10.18632/oncotarget.26652

**Published:** 2019-02-08

**Authors:** Sarwat Naz, John A. Cook, James B. Mitchell

**Affiliations:** Radiation Biology Branch, Center for Cancer Research, National Cancer Institute, Bethesda, MD, USA

**Keywords:** radiation, CDK4/6 inhibitor, abemaciclib, radiosensitizer, vasculogenesis

With recent development and FDA approval of highly specific CDK4/6 inhibitors (Palbociclib, Ribociclib, and Abemaciclib) for advanced metastatic breast cancer, with or without fulvestrant or letrozole have demonstrated very favorable tumor responses, making these drugs reasonable choices in appropriately selected patients. Early clinical successes have motivated clinicians to design multiple clinical trials with these agents in various solid tumors [1]. Amongst the three specific CDK4/6 inhibitors, Abemaciclib (Verzenio™) has demonstrated an impressive safety profile allowing for continuous oral dosing in various cancers [1, 2]. Worldwide, Abemaciclib is undergoing phase I–III trials for the treatment of breast cancer and non-small cell lung cancer (NSCLC), phase II development for the treatment of brain tumors, liposarcoma, mantle-cell lymphoma, colon cancer, pancreatic ductal adenocarcinoma, and preclinical development for several other solid tumors [1, 2]. Despite its success in phase I and II clinical trials, recent reports of Abemaciclib failure as monotherapy in a phase III trial for KRAS-mutant NSCLC has been discouraging (http://www.ascopost.com/News/58135). Abemaciclib did not meet its primary endpoint of overall survival in patients with KRAS-mutated, advanced NSCLC [3]. The failure of a specific cell cycle targeted agents to be effective as single agent anti-cancer drugs is not surprising; however, these types of drugs may hold the promise of being very effective when combined with other agents.

**Figure 1:**
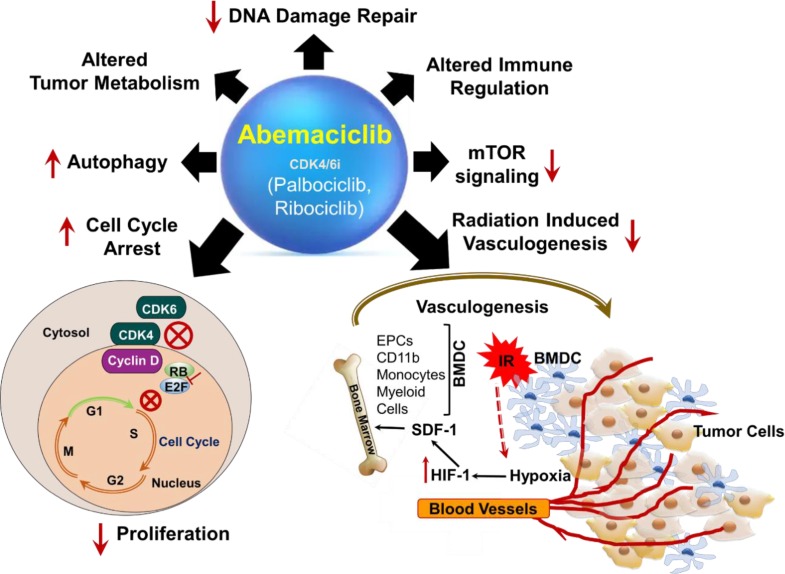
Abemaciclib, a selective CDK4/6 inhibitor with multiple actions CDK4/6 inhibitors (Abemaciclib, Palbociclib and Ribociclib) are cell cycle blockers mainly at the G1/S border. In the presence of these inhibitors, CDK4/6-cyclinD complex formation is inhibited resulting in reduced phosphorylation of RB (Retinoblastoma) protein and release of E2F transcription factor inside the nucleus to drive cells in the replicative (S) phase of cell cycle. Some numerous potential targets within the tumor microenvironment (TME) whose modulation may lead to radiosensitisation on tumor cells by Abemaciclib is highlighted and described in the text. Prior to irradiation, tumor growth is governed largely by local angiogenesis. When local angiogenesis is inhibited by irradiation, growth of the tumor vasculature (essential for recurrence of the tumor) can occur from circulating cells, of which BMDCs (Bone Marrow Derived Cells) such as CD11b+ monocytes, myeloid cells and EPCs (Endothelial Progenitors Cells) are an essential component. After irradiation, the tumor becomes more hypoxic, and HIF-1 (Hypoxia Inducible Factor-1) is increased as the tumor attempts to regrow. This induces SDF-1 (Stroma Derived Factor-1) and promotes the mobilization of CD11b+ monocytes from the BM (Bone Marrow) and retention of these BMDCs into the tumor. SDF-1 is the key factor for the influx of BMDC. Abemaciclib, inhibits restoration of tumor vasculature post radiotherapy by inhibiting HIF-1 and SDF-1 expression in the tumor microenvironment.

Interestingly, emerging pre-clinical studies have demonstrated that apart from their role in causing G1/S cell cycle arrest [1], cellular senescence and enhanced autophagy [4], CDK4/6 inhibitors can potentially impact several other cellular functions of both tumor (Figure 1) and normal cells. Abemaciclib has demonstrated multiple actions on tumor cells in different pre-clinical studies. It has been shown to elicit anti-tumor immune response leading to synergy with immune-checkpoint therapy [5] and alters tumor metabolism [2]. Another investigational CDK4/6 inhibitor, G1T28 protected against chemotherapy-induced bone marrow toxicity [6]. The excitement amongst oncologists to test these new CDK4/6 inhibitors in variety of solid tumors in combination with PI3K inhibitors and MEK inhibitors can be appreciated from the various ongoing clinical trials with these drugs [1-4].

Despite novel chemotherapy combinations in the clinics being explored with CDK4/6 inhibitors, it is important to emphasize the significance of chemoradiation received by cancer patients for local tumor control as well as for advanced metastatic disease. There has always been a growing interest amongst radiobiologists/radiation oncologists to identify new targeted agents that can be combined with radiotherapy to enhance the tumor therapeutic ratio. Recent published work from our group has demonstrated a paradigm shift in the role of Abemaciclib in combination with fractionated radiotherapy in NSCLC [7]. Our pre-clinical study indicated that amongst the three CDK4/6 inhibitors, only Abemaciclib could radiosensitize NSCLC cell lines independent of major oncogenic driver mutations in NSCLC namely, EGFR and RAS. However, RB dependency of tumors was clearly observed for this novel combination *in vitro*. The study demonstrated that Abemaciclib, in addition to inducing a G1/S phase cell cycle block, inhibited DNA damage repair and mTOR regulation, thus altering amino acid metabolism *in vitro*. Surprisingly, by administering Abemaciclib a second week post-radiation/Abemaciclib combination to lung cancer xenografts resulted in inhibition of radiation-induced neovascularization, also known as vasculogenesis *in vivo* and enhanced tumor growth delay [7].

Tumors support their growth by enhanced angiogenesis. Radiation has been shown to damage tumor vasculature and inhibit angiogenesis [8]. Tumor blood vessel restoration, and thus recurrence following radiation treatment occurs through a process of vasculogenesis [9]. This is so far considered the best mechanism to explain tumor recurrence post radiotherapy. Radiotherapy induced vasculogenesis was demonstrated in a series of elegant studies by Martin Brown's group in GBM mouse model where vasculogenesis as opposed to angiogenesis leads to vasculature recovery by colonization from bone marrow derived circulating cells (BMDC), primarily pro-angiogenic CD11b+ monocytes/macrophages. The stimulus for the influx of these CD11b+ cells into tumors following radiation is increased by enhanced levels of hypoxia inducible factor-1 (HIF-1) in the tumor due to induced tumor hypoxia secondary to blood vessel loss. This in turn leads to increased levels of the chemokine stromal cell-derived factor-1 (SDF-1), which binds to its receptors CXCR4 and CXCR7 expressed on monocytes and endothelial cells thereby trapping these cells in the tumor for making new blood vessels. This allows tumor cells with enough supply of nutrients and oxygen to further recur and resume growth [9].

Our study showed a unique role of Abemaciclib in inhibiting both HIF-1 and SDF-1 induction thereby mitigating radiation induced vasculogenesis. The findings that Abemaciclib enhanced tumor cell radiosensitivity, enhanced phosphorylation of gamma-H2AX in combination with radiation, reduced phosphorylation of p-AKT, p-S6 attenuating PI3K/mTOR signaling as well as alleviating radiation-induced vasculogenesis qualifies it to be a multi-functional radiation modifier (see Figure 1) [7]. The exciting aspect of this study is that it provides the framework to explore new mechanisms of action of CDK4/6 inhibition that were uncovered by combining Abemaciclib with radiation. For example, how does CDK4/6 inhibition alter radiation induced vasculogenesis and DNA damage repair? What is the mechanism of Abemaciclib mediated inhibition of HIF-1? What is the role of CDK4/6 inhibitors in the mobilization of BMDC to the irradiated site in the tumor? Does Abemaciclib impose a direct or indirect effect on the inhibition of SDF-1/CXCR4/CXCR7 interaction or are there secondary pathways involved? Answers to these questions await future research. Given the emerging role of radiation in immuno-oncology [10] it remains to be seen how Abemaciclib can change treatment outcomes of patients receiving radiotherapy for local tumor control. Given the specificity and low toxicity profile of Abemaciclib, combining this drug with radiation could possibly benefit lung cancer and other cancer patients receiving radiation as standard of care to not only increase local tumor control but to also lower their risk to recurrence post radiotherapy.
